# Forecasting and Responsible Innovation: A Book Review

**DOI:** 10.3389/fsoc.2022.835277

**Published:** 2022-05-18

**Authors:** António Brandão Moniz

**Affiliations:** Nova School of Sciences and Technology, University Nova of Lisbon, Lisbon, Portugal

**Keywords:** forecasting, responsible innovation, ethics, responsible research and innovation (RRI), future vision

## Abstract

The new book edited by Rodríguez and colleagues focuses on the topic of forecasting and responsible innovation. The original title is “Anticipación e Innovación Responsible: La construcción de futuros alternativos para la ciencia y la tecnologia” (Forecasting and Responsible Innovation: The construction of alternative futures for science and technology), and was published by Biblioteca Nueva, Madrid. Throughout this text, the reviewer is using the term *forecasting* instead of *anticipation* to convey the Spanish concept of “anticipación.” Both concepts are usually applied to “the act of looking forward” (Merriam-Webster dictionary^1^) or “the act of expecting or foreseeing something; expectation or presentiment” (Farlex free dictionary^2^) The concept of forecasting is usually used in scientific debate to mean “to estimate or predict in advance” (American Heritage Dictionary of the English Language, 2016) or “the process of making predictions based on past and present data and most commonly by analysis of trends” (Wikipedia^3^) (Glenn, 1994, p. 4) expressed this definition well by saying that “studying the future is not simply economic projections or sociological analysis or technological forecasting, but a multi-disciplinary examination of change in all major areas of life to find the interacting dynamics that are creating the next age.” The concept has been developed mainly by Armstrong (2001) and followed by Farrukh and Holgado (2020), Schnaars (2009), and Marinakis (2012), among others. The editors are professors and researchers from the University of Basque Country (EHU) and from the University of Mondragon (MU). The book involves a whole set of experts on the topic, including the editors themselves (Hannot Rodríguez, Sergio Urueña, Andoni Eizagirre, and Oier Imaz), and Armin Grunwald, René von Schomberg, Javier Garcia Fronti, Domingo García Marzá, Andoni Ibarra, and others. Although still published just in Spanish, it is an important contribution to the social sciences and philosophy of sciences regarding the analysis of alternative sociotechnical futures with strong ethical principles, which delineates an innovative approach in an era when the formation of public opinion largely suffers from systematic distortions based on vested interests.

## Structure of The Book

Forecasting has become, in recent decades, an important policy-making tool. Glenn (1994) refers to it by telling us that “the purpose of future studies is not to know the future but to make better decisions today.” Many authors established a wide diversity of methodological proposals to address foresight and anticipation analysis in relation to many scientific disciplines. Godet (1985) developed the scenario method to be applied to strategic management, Coates and Jarratt (1992) proposed that the debate should be applied to the political sciences while Carley and Bustelo (1984) critically reviewed a good number of social impact assessment studies, and many others presented new ideas. We can mention the studies on the environment forecasting that lead to the assumptions on climate change, the studies to model the macroeconomic trends, or the forecasts that enable visions on technology possibilities.

More recently, it became clear that policy planning modeling and decisions, or the technology assessment processes, need the framing of ethical principles that can govern the innovation processes. The book that we analyze here is about Forecasting and Responsible Innovation and integrating the main problems associated with these concepts.

The book is divided into three parts. The first one presents the theoretical fundamentals of forecasting and responsible innovation. The second one is mostly focused on the second mentioned concept: responsible innovation. The chapters approach the problem of how forecasting and inclusivity can contribute to responsible innovation. The third part is about practical applications.

The first part comprises four main chapters. They focus, respectively, on the paradox and role of forecasting on the history of science (Pérez Laraudogoitia, 2019), on the forecasting risks (Rodríguez and Urueña, 2019), on the responsiveness and “response-ability” toward techno-scientific forecasting (Arraiza Zabalegui, 2019), and on the definition of new technologies in the responsible research and innovation (RRI) processes (Grunwald, 2019).

The second part has three chapters. In the first chapter, von Schomberg (2019) develops the concept of “responsible innovation.” The next chapter, authored by Imaz et al. (2019) develops the idea of anticipatory governance in order to reach the aim of proposing the democratization of sociotechnical futures. Finally, this part concludes with a chapter on reflexivity as a means to stimulate RRI forecasting (González Esteban, 2019).

The third, and last, part of the book reveals three other chapters on practical contexts. The first one concerns the integration of ethics and communication on RRI management (Sanahuja et al., 2019). The second chapter discusses the subject of social laboratories as providing a space to disseminate RRI forecasting (Tabarés Gutiérrez and Bierwirth, 2019). The final chapter of this part, and of the whole book, is about big data technology forecasting governance, based on a case from Buenos Aires (García Fronti and Matías Herrera, 2019). The contribution is summarized in the next pages.

Starting with Pérez Laraudogoitia (2019, p. 55), he proposes the distinction between scientific modes of forecasting (based on predictions) and modes of scientific forecasting (metatheories about science), which can be the basis of an approach of forecasting context. He considers the institution of science as an anticipatory system with finalized purposes, which creates a coherent image of reality and of human experience.

In their contribution, Rodríguez and Urueña (2019, p. 84) provide an analysis of scientific and technology-related risks in the context of the EU innovation system. For them, *responsibility* is a function of the inclusivity degree of the research and innovation processes. At the same time, *risk* is the limiting element of that innovation system, in particular, examples can be framed by developments in nanotechnology, like atomic and molecular manipulations, with potential toxicity and biological reactivity. As they underline, “our proposal of ‘forecasting risk' could promote (radically) alternative modes of designing and complementing the milestones of socio-technic safety, beyond the imposed limits by a certain (here ‘objectivist') approach to the risk phenomenon, even in times of RRI” (p. 85).

The chapter by Arraiza Zabalegui, 2019, p. 121) aims to develop the concepts of responsibility and forecasting to enable responsiveness and responsibility. As the author explains, “the political-epistemic relevance of consequences and care help to conceptualize the problem with adequacy, which is essential to generate skillful answers for the present challenges”. The final idea can be “simple,” (as she admits) but is at the same time powerful, and it comes with the following words: “if we do not care, if we don't take care, if our discourses, practices and techno-scientific innovations do not start from-, with-, to- care, there will be no desirable, neither livable, futures to forecast” (p. 122).

Grunwald (2019) develops the idea of assigning meaning to the early characterization and debate around new and emergent technologies. This means the idea of “responsibility” must be clear at any initial step of such technological developments. For such purposes, it would be necessary to characterize the emergent technologies as novelties that should incorporate RRI. The assessment of those novelties can predict if each of those technologies is an evolution of previous stages or a revolutionary leap, with associated risks. Such definitions have consequences for ethical debates. They can also be inclusive or exclusive once they establish distinctions and limits. This can establish a social function as well as—conflicts when definitions grant certain groups access to work with new technologies and exclude others. Finally, Grunwald (2019) examines the choice of concepts and basic notions when definitions are established. The case of the prefix “nano” is one example, while the attribute “synthetic” for biology is another one. He concludes that a hermeneutic analysis is necessary, as is an assessment of the construction of the sociotechnical meaning of new technologies through definitions and feature descriptions.

The chapter on reasons for responsible innovation, from von Schomberg (2019), starts the second part of the book. The author points out six deficits that global research and innovation exemplify, which do not satisfy the desirable social objectives. They are the following:

1. Focus just on the risk and safety themes about new technologies, which enable mostly governmental regulations
2. Market insufficiencies toward desirable social innovations
3. Aligning innovation with public shared values and expectations
4. Interest for responsible technology development and technological potentials is higher than for responsible innovation
5. Absence of open research systems and an open academy (necessary but not sufficient condition for responsible innovation)
6. Lack of foresight and anticipatory governance for an alternative conformation of the sectoral innovation.

Schomberg concludes that a “meaningful foresight, the normative design of technology and the impact assessment can drive us towards the “correct impacts,” or positive results, of research and innovation” (p. 192) but that would require the aforementioned openness from academies and research activities to enable the development of socially desirable innovations.

The chapter from Imaz et al. (2019) starts with a detailed analysis of the concept of RRI. This can be a principle to the governance of “science with and for society.” They propose a systematic analysis for the “deliberative governance” of RRI. On this, the authors mention that the “open design of forecasting is a *necessary* but not *sufficient* condition for research and innovation governance based on the RRI principles” (p. 212). This brings to the debate the relation between democracy and RRI. On that, they use the contributions from Reber (2016, 2018), where deliberation is defined as an argumentative procedure that offers “the first opportunity to consider and experience that the normative evaluations of others can be different from theirs whilst still being rational” (Reber, 2016, p. 212). This can contribute to responding to uncertainty in research and innovation processes when providing ethical and epistemic pluralities. Finally, Imaz et al. (2019) articulate the concepts of RRI and democratic deliberation to develop the governance system of innovation.

The final text of this second part is written by González Esteban (2019) on reflexivity as a strategy to support forecasting on RRI. She uses references mostly from Lynch (2000) and Wynne (1993, 2002). Concerning the first case, she uses Lynch to affirm reflexivity should “reveal forgotten choices, expose hidden alternatives, lay bare epistemological limits and empower voices which had been subjugated by objective discourse” (Lynch, 2000, p. 36). This would facilitate capacity to the critical approach and to the emancipatory capacity of reflexivity, following the proposal of U. Beck: reflexivity should allow independence toward dominant science. From B. Wynne, González Esteban follows the definition of reflexivity proposed as “systematic processes of exploration of the prior commitments framing knowledge, in the way it has been introduced in sociological debates on modernity, rather than the more methodological-epistemological principle of consistency as it has been developed in the sociology of science” (Wynne, 1993, p. 321). She concludes that forecasting in RRI through reflexivity implies an approach from moral validity. This would enable ethical and critical reflexivity of RRI.

The final part of the book on practical contexts of responsible innovation and forecasting starts with a chapter from Sanahuja et al. (2019, p. 255) who propose the integration of ethics and communication dimensions on the RRI management processes. When interaction is a communication key for RRI, they proved through the results of experimental projects to enable alternatives for a communication model aligned with a “responsible science.” As they conclude, “a dialogical model of communication contributes to aligning processes and results with societal expectations and interests. The interaction with the interest groups increases the scientific culture of those that are participating” (p. 283).

The second chapter of this last part of the book is about social laboratories. The chapter is authored by Tabarés Gutiérrez and Bierwirth (2019), both at the large technology developer Tecnalia, and they also focus on practical contexts. They use the reference of a European project on social laboratories at the Horizon 2020 framework program^4^, which has studied 19 social labs that covered each of the areas of this program. The case study presented in this chapter is about the program Spreading Excellence and Widening Participation (SEWP). They have concluded about a dependency of the debate on RRI on the EU financing programs. Not all EU member states are involved in such a debate. It risks becoming relevant among only certain countries. That is considered a major challenge to the values that are elementary to the RRI concept. It risks being disseminated only among a certain group of privileged national communities.

The final chapter (of the book and of the section) is about Big Data technology forecasting governance (García Fronti and Matías Herrera, 2019). This study concluded that Big Data dynamics reveal privacy problems, which are particularly relevant when these technologies are used by the state. Thus, forecasting governance for these technologies becomes relevant. The case was developed for the Buenos Aires municipal government. This state institution wanted to develop stochastic governance using data on the population, but there was no problematization about the Big Data technologies. That municipal governance model would become difficult to maintain due to criticisms and regulation contradictions, and the project was suspended. The strategy had to be diverted to organize public debates on those technologies. This forecasting activity revealed that data are socially constructed. Thus, data are not a question of facts, but a question of interests. For this reason, it is important to bring data analysis to the public debate (p. 326).

## Limits and Criticism

This thematic collection of texts reflects an intensive debate in Spain, led by a large group of Basque Country researchers on ethics and philosophy of science and technology. That is an important achievement that can be brought to the wider public. The theme of RRI management is no longer a debated concept confined to the central European countries (and more recently, to the US), but is wider. It has been developed in several universities of Spain, as well as in other countries.

However, the forecasting governance and the responsibility dimension of research and innovation have been brought to the public debate mostly by philosophers and ethicists. If it does not involve other types of scientific expertise, it might become an isolated debate. Some of the chapters of this important book raised that issue. But this can be one of the main criticisms of the work edited by Rodríguez, Urueña, Eizagirre, and Imaz. It seems to be difficult to bring around the conceptual discussion and open a dialog with the epistemological framework of other disciplines, arguably, sociology and political sciences, anthropology, economics, and management sciences. The debate on responsible research and innovation is not exclusively addressing the epistemological frameworks of philosophy. Such debate proves the need for interdisciplinary analytical tools, and for other contributions from social sciences. This can be done with articles organized and written by experts from different disciplines in an interdisciplinary mode, for example, with thematic collective works. Most probably, new insights can be taken, and new critical perspectives may be raised and that would contribute to renewing the debate.

Although that criticism may be raised, the value of the book cannot be diminished. It is a comparable contribution to the major English-speaking works recently published around the world. Being published only in Spanish is not a limitation. Because of that, it can bring the discussion to the wider Latin-American academic world. Some chapters with examples from South America could prove this. This means as well that more publication of valuable texts like these becomes a (public) need. On that, the authors from Basque Country prove that they can lead the way they have paved on this topic.

## A Debate to Follow

The debate has been settled by several recent publications on RRI and its governance processes. But from the description we have made above, and the information we got from other publications, it needs the inclusion of more perspectives from social sciences. It is needs to approach more research communities and national traditions. This was analyzed in one chapter (Tabarés Gutiérrez and Bierwirth, 2019), but the observation of major publications also reveals the urgent need to involve more experts and experiences. These would not be diversified only in terms of nationalities but also in terms of scientific disciplines.

If such debate has only been developed among a few academics, it risks losing its main value: responsibility.

## Author Contributions

The author confirms being the sole contributor of this work and has approved it for publication.

## Conflict of Interest

The author declares that the research was conducted in the absence of any commercial or financial relationships that could be construed as a potential conflict of interest.

## Publisher's Note

All claims expressed in this article are solely those of the authors and do not necessarily represent those of their affiliated organizations, or those of the publisher, the editors and the reviewers. Any product that may be evaluated in this article, or claim that may be made by its manufacturer, is not guaranteed or endorsed by the publisher.

## Footnotes

^4^Project New HoRRIzon http://newhorrizon.eu.
